# Supplementary material for: Drivers of HIV-1 transmission: The Portuguese case
Source: PLoS One. 2019 Sep 30;14(9):e0218226. doi: 10.1371/journal.pone.0218226 (PMC6768452; doi:10.1371/journal.pone.0218226)
Supplement: S1 File — Table A: Characteristics of the subtype B population in the PT-naive cohort between 2001 and 2014. Missing data was less than 5%, the exception were variables with an asterisk. CD4 count, risk of transmission and continent of origin were excluded from the multivariate analyses given the amount of missing data. Abbreviations: IQR: interquartile range, n: sample, TDR: transmitted drug resistance, % percentage. Table B: Characteristics of the subtype G population in the PT-naive cohort between 2001 and 2014. Missing data was less than 5%, the exception were variables with an asterisk. CD4 count, risk of transmission and continent of origin were excluded from the multivariate analyses given the amount of missing data. Abbreviations: IQR: interquartile range, n: sample, TDR: transmitted drug resistance, % percentage. Table C: Characteristics of the “pure” subtype G population in the PT-naive cohort between 2001 and 2014. Missing data was less than 5%, the exception were variables with an asterisk. CD4 count, risk of transmission and continent of origin were excluded from the multivariate analyses given the amount of missing data. Abbreviations: IQR: interquartile range, n: sample, TDR: transmitted drug resistance, % percentage. Table D:Table D. Characteristics of the transmission clusters (TCs) in the PT-naive cohort and in the PT-naive cohort with TDR for the “pure” subtype G. Abbreviations: IQR: interquartile range, n: sample, NS: No significant, TCs: Transmission clusters TDR: transmitted drug resistance, % percentage. (DOCX) [file pone.0218226.s001.docx]

**S1 File**

**Drivers of HIV-1 transmission: the Portuguese case**

**Running title:** Drivers of HIV-1 transmission in Portugal

**Characteristics of the PT-naive cohort**

**Table A:** Characteristics of the subtype B population in the PT-naive cohort between 2001 and 2014. Missing data was less than 5%, the exception were variables with an asterisk. CD4 count, risk of transmission and continent of origin were excluded from the multivariate analyses given the amount of missing data. Abbreviations: IQR: interquartile range, n: sample, TDR: transmitted drug resistance, % percentage.

|  | **Subtype B** | | | | | | | | | | | | | | | | | | | | | | | | | | | |  |
| --- | --- | --- | --- | --- | --- | --- | --- | --- | --- | --- | --- | --- | --- | --- | --- | --- | --- | --- | --- | --- | --- | --- | --- | --- | --- | --- | --- | --- | --- |
| **Characteristics at time of sampling** | **Total** | | | **Cohort with TDR** | | | **Cohort in clusters** | | | | | | **TDR clusters** | | | | | **Clusters ≥3** | | | | **TDR clusters ≥3** | | **Active clusters** | | **Onward TDR clusters** | | | |
|  |  |  |  |  |  |  |  |  |  |  |  |  |  |  |  |  |  |  |  |  |  |  |  |  |  |  |  |  |  |
|  | **n** | | **%** | **n** | **%** | | | **n** | | **%** | | | | **n** | | **%** | | **n** | | **%** | | **n** | **%** | **n** | **%** | | **n** | **%** | |
| **Total number of patients** | | 2042 |  | 221 |  | 1250 | | | | |  | | | | 146 | |  | | 828 | |  | 99 |  | 286 |  | | 74 |  | |
| **Male** | | 1592 | 78.3 | 172 | 78.2 | 1004 | | | | | 80.8 | | | | 118 | | 81.4 | | 707 | | 85.8 | 83 | 84.7 | 253 | 88.5 | | 59 | 80.8 | |
|  |  | |  |  |  |  | | |  | | |  | | | |  | |  | | |  |  |  |  |  | |  |  | |
| **Age in years at enrolment, Median (IQR)** | 37 | | (30-45) | 35 | (30-45) | 36 | | | (29-44) | | | 35 | | | | (29-44) | | 35 | | | (28-43) | 33 | (27-40) | 34 | (26-42) | | 34 | (30-38) | |
| <25 | 191 | | 9.8 | 20 | 9.5 | 150 | | | 12.6 | | | 17 | | | | 12.2 | | 113 | | | 14.3 | 14 | 14.6 | 47 | 17.2 | | 8 | 11.1 | |
| 25-34 | 591 | | 30.4 | 78 | 37.1 | 378 | | | 31.7 | | | 52 | | | | 37.4 | | 265 | | | 33.5 | 40 | 41.7 | 94 | 34.4 | | 31 | 43.1 | |
| 35-44 | 644 | | 33.1 | 58 | 27.6 | 368 | | | 30.9 | | | 34 | | | | 24.5 | | 238 | | | 30.1 | 21 | 21.9 | 80 | 29.3 | | 19 | 26.4 | |
| 45-54 | 352 | | 18.1 | 35 | 16.7 | 192 | | | 16.1 | | | 22 | | | | 15.8 | | 121 | | | 15.3 | 14 | 14.6 | 35 | 12.8 | | 9 | 12.5 | |
| >55 | 169 | | 8.7 | 19 | 9.0 | 104 | | | 8.7 | | | 14 | | | | 10.1 | | 54 | | | 6.8 | 7 | 7.3 | 17 | 6.2 | | 5 | 6.9 | |
|  |  | |  |  |  |  | | |  | | |  | | | |  | |  | | |  |  |  |  |  | |  |  | |
| **Region of Residence** |  | |  |  |  |  | | |  | | |  | | | |  | |  | | |  |  |  |  |  | |  |  | |
| Alentejo | 39 | | 1.9 | 6 | 2.8 | 20 | | | 1.6 | | | 3 | | | | 2.1 | | 12 | | | 1.5 | 1 | 1.0 | 3 | 1.1 | | 0 | 0.0 | |
| Algarve | 214 | | 10.6 | 27 | 12.4 | 135 | | | 11.0 | | | 18 | | | | 12.7 | | 80 | | | 9.9 | 9 | 9.4 | 44 | 16.1 | | 4 | 5.6 | |
| Center | 87 | | 4.3 | 16 | 7.4 | 55 | | | 4.5 | | | 10 | | | | 7.0 | | 36 | | | 4.4 | 7 | 7.3 | 9 | 3.3 | | 6 | 8.5 | |
| Islands | 67 | | 3.3 | 4 | 1.8 | 57 | | | 4.6 | | | 10 | | | | 2.8 | | 45 | | | 5.6 | 4 | 4.2 | 25 | 9.1 | | 2 | 2.8 | |
| Lisboa e Vale do Tejo | 1600 | | 79.5 | 163 | 75.1 | 957 | | | 77.8 | | | 10 | | | | 74.6 | | 635 | | | 78.4 | 75 | 78.1 | 192 | 70.1 | | 59 | 83.1 | |
| North | 6 | | 0.3 | 1 | 0.5 | 6 | | | 0.5 | | | 10 | | | | 0.7 | | 2 | | | 0.2 | 0 | 0.0 | 1 | 0.4 | | 0 | 0.0 | |
|  |  | |  |  |  |  | | |  | | |  | | | |  | |  | | |  |  |  |  |  | |  |  | |
| **Continent of origin*** |  | |  |  |  |  | | |  | | |  | | | |  | |  | | |  |  |  |  |  | |  |  | |
| Portugal | 766 | | 64.8 | 77 | 62.6 | 501 | | | 68.6 | | | 55 | | | | 68.8 | | 357 | | | 72.6 | 41 | 82.0 | 136 | 81.9 | | 30 | 76.9 | |
| Other European countries | 249 | | 21.1 | 23 | 18.7 | 146 | | | 20.0 | | | 15 | | | | 18.8 | | 86 | | | 17.5 | 7 | 14.0 | 6 | 3.6 | | 7 | 17.9 | |
| Africa | 70 | | 5.9 | 10 | 8.1 | 33 | | | 4.5 | | | 5 | | | | 6.3 | | 19 | | | 3.9 | 1 | 2.0 | 4 | 2.4 | | 1 | 2.6 | |
| America | 93 | | 7.9 | 12 | 9.8 | 49 | | | 6.7 | | | 5 | | | | 6.3 | | 30 | | | 6.1 | 1 | 2.0 | 20 | 12.0 | | 1 | 2.6 | |
| Asia | 4 | | 0.3 | 1 | 0.8 | 1 | | | 0.1 | | | 0 | | | | 0.0 | | 0 | | | 0.0 | 0 | 0.0 | 0 | 0.0 | | 0 | 0.0 | |
|  |  | |  |  |  |  | | |  | | |  | | | |  | |  | | |  |  |  |  |  | |  |  | |
| **Risk of transmission*** |  | |  |  |  |  | | |  | | |  | | | |  | |  | | |  |  |  |  |  | |  |  | |
| MSM/Bisexual | 113 | | 47.7 | 14 | 70.0 | 79 | | | 53.7 | | | 12 | | | | 70.6 | | 58 | | | 61.7 | 8 | 80.0 | 22 | 68.8 | | 5 | 100.0 | |
| Heterosexual | 82 | | 34.6 | 2 | 10.0 | 48 | | | 32.7 | | | 2 | | | | 11.8 | | 25 | | | 26.6 | 0 | 0.0 | 9 | 28.1 | | 0 | 0.0 | |
| IVDU | 41 | | 17.3 | 4 | 20.0 | 20 | | | 13.6 | | | 3 | | | | 17.6 | | 11 | | | 11.7 | 2 | 20.0 | 1 | 3.1 | | 0 | 0.0 | |
| Other | 1 | | 0.4 | 0 | 0.0 | 0 | | | 0.0 | | | 0 | | | | 0.0 | | 0 | | | 0.0 | 0 | 0.0 | 0 | 0.0 | | 0 | 0.0 | |
|  |  | |  |  |  |  | | |  | | |  | | | |  | |  | | |  |  |  |  |  | |  |  | |
| **CD4 cell count, median (IQR)*** | 333 | | (139-512) | 368 | (225-562) | 364 | | | (190-525) | | | 378 | | | | (271-555) | | 367 | | | (193-560) | 376 | (273-618) | 436 | (282-570) | | 387 | (285-587) | |
| <200 cells/mm3 | 314 | | 31.2 | 26 | 23.2 | 162 | | | 26.7 | | | 14 | | | | 18.9 | | 104 | | | 25.7 | 9 | 16.7 | 23 | 15.6 | | 5 | 13.2 | |
| 200-349 cells/mm3 | 210 | | 20.9 | 22 | 19.6 | 125 | | | 20.6 | | | 17 | | | | 23.0 | | 83 | | | 20.5 | 13 | 24.1 | 28 | 19.0 | | 9 | 23.7 | |
| 350-499 cells/mm3 | 222 | | 22.0 | 30 | 26.8 | 150 | | | 24.7 | | | 21 | | | | 28.4 | | 91 | | | 22.5 | 15 | 27.8 | 40 | 27.2 | | 13 | 34.2 | |
| ≥500 | 261 | | 25.9 | 34 | 30.4 | 170 | | | 28.0 | | | 22 | | | | 29.7 | | 127 | | | 31.4 | 17 | 31.5 | 56 | 38.1 | | 11 | 28.9 | |
|  |  | |  |  |  |  | | |  | | |  | | | |  | |  | | |  |  |  |  |  | |  |  | |
| **HIV-RNA load, Log10 copies/ml, median (IQR)*** | 4.78 | | (4.22-5.30) | 4.56 | (4.03-5.17) | 4.78 | | | (4.22-5.28) | | | 4.56 | | | | (4.08-5.16) | | 4.81 | | | (4.29-5.32) | 4.71 | (4.22-5.22) | 4.83 | 4.38-5.31 | | 4.75 | (4.24-5.17) | |

**Table B:** Characteristics of the subtype G population in the PT-naive cohort between 2001 and 2014. Missing data was less than 5%, the exception were variables with an asterisk. CD4 count, risk of transmission and continent of origin were excluded from the multivariate analyses given the amount of missing data. Abbreviations: IQR: interquartile range, n: sample, TDR: transmitted drug resistance, % percentage.

|  | **Subtype G** | | | | | | | | | | | | | | | |
| --- | --- | --- | --- | --- | --- | --- | --- | --- | --- | --- | --- | --- | --- | --- | --- | --- |
| **Characteristics at time of sampling** | **Total** | | **Cohort with TDR** | | **Cohort in clusters** | | **TDR clusters** | | **Clusters ≥3** | | **TDR clusters ≥3** | | **Active clusters** | | **Onward TDR clusters** | |
|  |  |  |  |  |  |  |  |  |  |  |  |  |  |  |  |  |
|  | **n** | **%** | **n** | **%** | **n** | **%** | **n** | **%** | **n** | **%** | **n** | **%** | **n** | **%** | **n** | **%** |
| **Total number of patients** | 1557 |  | 118 |  | 726 |  | 51 |  | 411 |  | 30 |  | 93 |  | 22 |  |
| **Male** | 896 | 58.1 | 68 | 58.6 | 393 | 54.6 | 29 | 58.0 | 217 | 52.8 | 16 | 55.2 | 40 | 43.0 | 14 | 66.7 |
|  |  |  |  |  |  |  |  |  |  |  |  |  |  |  |  |  |
| **Age in years at enrolment, Median (IQR)** | 38 | (32-47) | 40 | (33-52) | 38 | (31-47) | 44 | (34-54) | 39 | (31-48) | 44 | (34-54) | 36 | (29-43) | 44 | (35-49) |
| <25 | 105 | 7.1 | 7 | 6.4 | 70 | 10.1 | 4 | 8.2 | 45 | 10.9 | 3 | 10.7 | 17 | 18.9 | 3 | 15.0 |
| 25-34 | 423 | 28.6 | 28 | 25.7 | 193 | 27.8 | 9 | 18.4 | 104 | 25.3 | 4 | 14.3 | 22 | 24.4 | 1 | 5.0 |
| 35-44 | 485 | 32.8 | 32 | 29.4 | 221 | 31.8 | 15 | 30.6 | 117 | 28.5 | 9 | 32.1 | 31 | 34.4 | 8 | 40.0 |
| 45-54 | 268 | 18.1 | 20 | 18.3 | 112 | 16.1 | 9 | 18.4 | 64 | 15.6 | 5 | 17.9 | 15 | 16.7 | 4 | 20.0 |
| >55 | 198 | 13.4 | 22 | 20.2 | 98 | 14.1 | 12 | 24.5 | 66 | 16.1 | 7 | 25.0 | 5 | 5.6 | 4 | 20.0 |
|  |  |  |  |  |  |  |  |  |  |  |  |  |  |  |  |  |
| **Region of Residence** |  |  |  |  |  |  |  |  |  |  |  |  |  |  |  |  |
| Alentejo | 37 | 2.4 | 2 | 1.7 | 14 | 1.9 | 1 | 2.0 | 10 | 2.4 | 1 | 3.4 | 0 | 0.0 | 0 | 0.0 |
| Algarve | 179 | 11.6 | 21 | 18.3 | 82 | 11.4 | 10 | 20.4 | 37 | 9.0 | 9 | 31.0 | 20 | 21.7 | 10 | 47.6 |
| Center | 89 | 5.8 | 13 | 11.3 | 33 | 4.6 | 4 | 8.2 | 13 | 3.2 | 2 | 6.9 | 1 | 1.1 | 2 | 9.5 |
| Islands | 11 | 0.7 | 1 | 0.9 | 6 | 0.8 | 0 | 0.0 | 5 | 1.2 | 0 | 0.0 | 2 | 2.2 | 0 | 0.0 |
| Lisboa e Vale do Tejo | 1224 | 79.1 | 77 | 67.0 | 584 | 81.0 | 34 | 69.4 | 343 | 83.5 | 17 | 58.6 | 69 | 75.0 | 9 | 42.9 |
| North | 7 | 0.5 | 1 | 0.9 | 2 | 0.3 | 0 | 0.0 | 0 | 0.0 | 0 | 0.0 | 0 | 0.0 | 0 | 0.0 |
|  |  |  |  |  |  |  |  |  |  |  |  |  |  |  |  |  |
| **Continent of origin*** |  |  |  |  |  |  |  |  |  |  |  |  |  |  |  |  |
| Portugal | 543 | 61.9 | 39 | 66.1 | 274 | 67.7 | 20 | 74.1 | 163 | 39.7 | 11 | 84.6 | 41 | 85.4 | 10 | 90.9 |
| Other European countries | 191 | 21.8 | 15 | 25.4 | 72 | 17.8 | 5 | 18.5 | 31 | 7.5 | 1 | 7.7 | 2 | 4.2 | 1 | 9.1 |
| Africa | 125 | 14.3 | 3 | 5.1 | 50 | 12.3 | 1 | 3.7 | 30 | 7.3 | 1 | 7.7 | 4 | 8.3 | 0 | 0.0 |
| America | 15 | 1.7 | 1 | 1.7 | 7 | 1.7 | 1 | 3.7 | 4 | 1.0 | 0 | 0.0 | 0 | 0.0 | 0 | 0.0 |
| Asia | 3 | 0.3 | 1 | 1.7 | 2 | 0.5 | 0 | 0.0 | 0 | 0.0 | 0 | 0.0 | 1 | 2.1 | 0 | 0.0 |
|  |  |  |  |  |  |  |  |  |  |  |  |  |  |  |  |  |
| **Risk of transmission*** |  |  |  |  |  |  |  |  |  |  |  |  |  |  |  |  |
| MSM/Bisexual | 13 | 6.8 | 1 | 6.7 | 8 | 9.6 | 0 | 0.0 | 3 | 0.7 | 0 | 0.0 | 1 | 12.5 | 0 | 0.0 |
| Heterosexual | 89 | 46.4 | 11 | 73.3 | 39 | 47.0 | 4 | 66.7 | 22 | 5.4 | 2 | 66.7 | 3 | 37.5 | 2 | 50.0 |
| IVDU | 89 | 46.4 | 3 | 20.0 | 35 | 42.2 | 2 | 33.3 | 19 | 4.6 | 1 | 33.3 | 4 | 50.0 | 2 | 50.0 |
| Other | 1 | 0.5 | 0 | 0.0 | 1 | 1.2 | 0 | 0.0 | 0 | 0.0 | 0 | 0.0 | 0 | 0.0 | 0 | 0.0 |
|  |  |  |  |  |  |  |  |  |  |  |  |  |  |  |  |  |
| **CD4 cell count, median (IQR)*** | 257 | (113-416) | 246 | (135-422) | 275 | (142-433) | 387 | (126-418) | 283 | (149-462) | 565 | (180-682) | 344 | 148-459 | 400 | (174-652) |
| <200 cells/mm3 | 297 | 40.4 | 22 | 41.5 | 121 | 36.7 | 8 | 42.1 | 67 | 16.3 | 3 | 33.3 | 19 | 36.5 | 2 | 33.3 |
| 200-349 cells/mm3 | 179 | 24.3 | 11 | 20.8 | 88 | 26.7 | 1 | 5.3 | 47 | 11.4 | 1 | 11.1 | 13 | 25.0 | 1 | 16.7 |
| 350-499 cells/mm3 | 135 | 18.3 | 8 | 15.1 | 59 | 17.9 | 2 | 10.5 | 34 | 8.3 | 0 | 0.0 | 10 | 19.2 | 0 | 0.0 |
| ≥500 | 125 | 17.0 | 12 | 22.6 | 62 | 18.8 | 8 | 42.1 | 40 | 9.7 | 5 | 55.6 | 10 | 19.2 | 3 | 50.0 |
|  |  |  |  |  |  |  |  |  |  |  |  |  |  |  |  |  |
| **HIV-RNA load, Log10 copies/ml, median (IQR)*** | 4.86 | (4.25-5.40) | 4.78 | (4.20-5.28) | 4.81 | (4.26-5.40) | 4.90 | (4.40-5.71) | 4.80 | (4.27-5.40) | 4.85 | (4.27-5.92) | 4.89 | (4.44-5.37) | 5.19 | (4.47-6.14) |

**Sensitivity analyses of different genetic distances and boostrap support**

Sensitivity analyses were performed with varying genetic distances (0.015, 0.030, 0.045, 0.060) and bootstrap supports (70, 90, 95, 98).

When considered TCs with different thresholds, age and male were consistently significantly associated with transmission of subtype B even when the strict threshold of 0.015 was considered, while none of the socio-demographic factors were associated with transmission of TDR.

Subtype G results remained similar.

**Sensitivity analysis using the definition of “pure” subtype G, excluding CRF14_BG**

The overall TDR prevalence was 7.8% for subtype G [79/1006, 6.3-9.6], 1.8% [19/1006; 1.2-2.9] for NRTI and PI and 5.1% [52/1006; 3.9-6.7] for NNRTI. A decreasing trend of TDR for NRTI was observed [p=0.003] in this subtype, which was significant even when the first period 2001-2002 was excluded [p=0.03]. None of the socio-demographic or clinical factors were significantly associated with TDR when compared with the population without TDR within subtype G. There was a decreasing trend of transmission of subtype G from 2005 in Portuguese people (p=0.01).

When compared transmission of subtype B versus G and only clusters ≥3, age and male were also significant in the multivariate analyses, but Europeans were also more likely to transmit subtype B (OR 2.11, 1.11-3.98, p=0.02). The rest of the analyses were like the ones presented for dataset G/CFR14_BG (Tables C and D in S1 text)

**Table C:** Characteristics of the “pure” subtype G population in the PT-naive cohort between 2001 and 2014. Missing data was less than 5%, the exception were variables with an asterisk. CD4 count, risk of transmission and continent of origin were excluded from the multivariate analyses given the amount of missing data. Abbreviations: IQR: interquartile range, n: sample, TDR: transmitted drug resistance, % percentage.

|  | **“Pure” Subtype G** | | | | | | | | | | | | | | | | | | | | | | | | | | | |  |
| --- | --- | --- | --- | --- | --- | --- | --- | --- | --- | --- | --- | --- | --- | --- | --- | --- | --- | --- | --- | --- | --- | --- | --- | --- | --- | --- | --- | --- | --- |
| **Characteristics at time of sampling** | **Total** | | | **Cohort with TDR** | | | **Cohort in clusters** | | | | **TDR clusters** | | | | **Clusters ≥3** | | **TDR clusters ≥3** | | | | **Active clusters** | | | | **Onward TDR clusters** | | | |  |
|  |  |  |  |  |  |  |  |  |  |  |  |  |  |  |  |  |  |  |  |  |  |  |  |  |  |  |  |  |  |
|  | **n** | **%** | | **n** | | **%** | **n** | **%** | | **n** | | | **%** | **n** | | **%** | | **n** | **%** | | | **n** | | **%** | | **n** | **%** | |  |
| **Patients** | 1006 | |  | 79 | |  | 464 |  | | 33 | | |  | 273 | |  | | 21 |  | | | 60 | |  | | 15 |  | |  |
| **Male** | 559 | 56.1 | | 43 | 55.8 | | 246 | 53.4 | 17 | | | 53.1 | | 140 | | 51.7 | | 11 | | 52.4 | | 34 | 57.6 | | | 9 | 60.0 | |  |
|  |  |  | |  |  | |  |  |  | | |  | |  | |  | |  | |  | |  |  | | |  |  | |  |
| **Age in years at enrolment, Median (IQR)** | 39 | (32-49) | | 42 | (32-52) | | 39 | (31-49) | 44 | | | (37-53)* | | 40 | | (31-50) | | 44 | | (37-50) | | 34 | (28-40) | | | 44 | (36-46) | |  |
| <25 | 75 | 7.8 | | 6 | 8.1 | | 48 | 10.8 | 4 | | | 12.9 | | 31 | | 11.8 | | 3 | | 15.8 | | 11 | 18.6 | | | 3 | 21.4 | |  |
| 25-34 | 255 | 26.6 | | 18 | 24.3 | | 112 | 25.2 | 3 | | | 9.7 | | 65 | | 24.8 | | 1 | | 5.3 | | 19 | 32.2 | | | 0 | 0.0 | |  |
| 35-44 | 285 | 29.7 | | 20 | 27.0 | | 133 | 29.9 | 9 | | | 29.0 | | 68 | | 26.0 | | 8 | | 42.1 | | 17 | 28.8 | | | 7 | 50.0 | |  |
| 45-54 | 192 | 20.0 | | 16 | 21.6 | | 79 | 17.8 | 8 | | | 25.8 | | 48 | | 18.3 | | 3 | | 15.8 | | 8 | 13.6 | | | 2 | 14.3 | |  |
| >55 | 151 | 15.8 | | 14 | 18.9 | | 73 | 16.4 | 7 | | | 22.6 | | 50 | | 19.1 | | 4 | | 21.1 | | 4 | 6.8 | | | 2 | 14.3 | |  |
|  |  |  | |  |  | |  |  |  | | |  | |  | |  | |  | |  | |  |  | | |  |  | |  |
| **Region of Residence** |  |  | |  |  | |  |  |  | | |  | |  | |  | |  | |  | |  |  | | |  |  | |  |
| Alentejo | 19 | 1.9 | | 0 | 0.0 | | 10 | 2.2 | 0 | | | 0.0 | | 9 | | 3.3 | | 0 | | 0.0 | | 0 | 0.0 | | | 0 | 0.0 | |  |
| Algarve | 135 | 13.5 | | 15 | 19.2 | | 61 | 13.2 | 7 | | | 21.9 | | 23 | | 8.5 | | 4 | | 20.0 | | 12 | 20.0 | | | 4 | 28.6 | |  |
| Center | 60 | 6.0 | | 8 | 10.3 | | 24 | 5.2 | 2 | | | 6.3 | | 12 | | 4.4 | | 2 | | 10.0 | | 1 | 1.7 | | | 2 | 14.3 | |  |
| Islands | 9 | 0.9 | | 1 | 1.3 | | 3 | 0.7 | 0 | | | 0.0 | | 2 | | 0.7 | | 0 | | 0.0 | | 1 | 1.7 | | | 0 | 0.0 | |  |
| Lisboa e Vale do Tejo | 776 | 77.6 | | 54 | 69.2 | | 363 | 78.7 | 23 | | | 71.9 | | 225 | | 83.0 | | 14 | | 70.0 | | 46 | 76.7 | | | 8 | 57.1 | |  |
| North | 1 | 0.1 | | 0 | 0.0 | | 0 | 0.0 | 0 | | | 0.0 | | 0 | | 0.0 | | 0 | | 0.0 | | 0 | 0.0 | | | 0 | 0.0 | |  |
|  |  |  | |  |  | |  |  |  | | |  | |  | |  | |  | |  | |  |  | | |  |  | |  |
| **Continent of origin*** |  |  | |  |  | |  |  |  | | |  | |  | |  | |  | |  | |  |  | | |  |  | |  |
| Portugal | 343 | 59.1 | | 20 | 57.1 | | 172 | 65.9 | 10 | | | 71.4 | | 111 | | 73.5 | | 8 | | 88.9 | | 25 | 80.6 | | | 6 | 85.7 | |  |
| Other European countries | 117 | 20.2 | | 11 | 31.4 | | 45 | 17.2 | 3 | | | 21.4 | | 15 | | 9.9 | | 1 | | 11.1 | | 2 | 6.5 | | | 1 | 14.3 | |  |
| Africa | 106 | 18.3 | | 2 | 5.7 | | 37 | 14.2 | 0 | | | 0.0 | | 22 | | 14.6 | | 0 | | 0.0 | | 3 | 9.7 | | | 0 | 0.0 | |  |
| America | 11 | 1.9 | | 1 | 2.9 | | 5 | 1.9 | 1 | | | 7.1 | | 3 | | 2.0 | | 0 | | 0.0 | | 0 | 0.0 | | | 0 | 0.0 | |  |
| Asia | 3 | 0.5 | | 1 | 2.9 | | 2 | 0.8 | 0 | | | 0.0 | | 0 | | 0.0 | | 0 | | 0.0 | | 1 | 3.2 | | | 0 | 0.0 | |  |
|  |  |  | |  |  | |  |  |  | | |  | |  | |  | |  | |  | |  |  | | |  |  | |  |
| **Risk of transmission*** |  |  | |  |  | |  |  |  | | |  | |  | |  | |  | |  | |  |  | | |  |  | |  |
| MSM/Bisexual | 6 | 4.8 | | 0 | 0.0 | | 4 | 7.7 | 0 | | | 0.0 | | 2 | | 6.9 | | 2 | | 100.0 | | 0 | 0.0 | | | 0 | 0.0 | |  |
| Heterosexual | 67 | 53.6 | | 7 | 87.5 | | 27 | 51.9 | 2 | | | 100.0 | | 16 | | 55.2 | | 0 | | 0.0 | | 2 | 40.0 | | | 2 | 100.0 | |  |
| IVDU | 51 | 40.8 | | 1 | 12.5 | | 20 | 38.5 | 0 | | | 0.0 | | 11 | | 37.9 | | 0 | | 0.0 | | 3 | 60.0 | | | 0 | 0.0 | |  |
| Other | 1 | 0.8 | | 0 | 0.0 | | 1 | 1.9 | 0 | | | 0.0 | | 0 | | 0.0 | | 0 | | 0.0 | | 0 | 0.0 | | | 0 | 0.0 | |  |
|  |  |  | |  |  | |  |  |  | | |  | |  | |  | |  | |  | |  |  | | |  |  | |  |
| **CD4 cell count,**  **median (IQR)*** | 250 | (113-404) | | 310 | (240-392) | | 274 | (142-416) | 236 | | | (114-631) | | 288 | | (146-450) | | 600 | | (185-672) | | 301 | (134-461) | | | 506 | (418-641) | | |
| <200 cells/mm3 | 193 | 41.3 | | 16 | 43.2 | | 80 | 36.7 | 7 | | | 46.7 | | 44 | | 35.5 | | 2 | | 28.6 | | 14 | 38.9 | | | 0 | 0.0 |  |  |
| 200-349 cells/mm3 | 116 | 24.8 | | 9 | 24.3 | | 56 | 25.7 | 2 | | | 13.3 | | 29 | | 23.4 | | 1 | | 14.3 | | 8 | 22.2 | | | 1 | 33.3 |  |  |
| 350-499 cells/mm3 | 85 | 18.2 | | 4 | 10.8 | | 41 | 18.8 | 1 | | | 6.7 | | 25 | | 20.2 | | 0 | | 0.0 | | 7 | 19.4 | | | 0 | 0.0 |  |  |
| ≥500 | 73 | 15.6 | | 8 | 21.6 | | 41 | 18.8 | 5 | | | 33.3 | | 26 | | 21.0 | | 4 | | 57.1 | | 7 | 19.4 | | | 2 | 66.7 |  |  |
|  |  |  | |  |  | |  |  |  | | |  | |  | |  | |  | |  | |  |  | | |  |  |  |  |
| **HIV-RNA load, Log10 copies/ml, median (IQR)*** | 4.86 | (4.25-5.40) | | 4.91 | (4.22-5.38) | | 4.77 | (4.25-5.39) | 5.17 | | | (4.36-5.73) | | 4.75 | | (4.27-5.38) | | 4.05 | | (4.23-5.72) | | 4.79 | (4.37-5.27) | | | 5.25 | (4.42-5.78) |  |  |

**Table D:** Characteristics of the transmission clusters (TCs) in the PT-naive cohort and in the PT-naive cohort with TDR for the “pure” subtype G. Abbreviations: IQR: interquartile range, n: sample, NS: No significant, TCs: Transmission clusters TDR: transmitted drug resistance, % percentage.

| **Characteristic** | **General cohort** | | | |  | **TDR cohort in TCs** | | | |  |
| --- | --- | --- | --- | --- | --- | --- | --- | --- | --- | --- |
|  | **B** | | **“pure” G** | | **B vs G** | **B** | | **“pure” G** | | **B vs “pure” G** |
|  | **n** | % | **n** | **%** | **p-value** | **n** | % | **n** | **%** | **p-value** |
| **Clusters** |  |  |  |  |  |  |  |  |  |  |
| **Number of cohort clusters** | 497 | 100 | 221 | 100 |  | 82 | 100 | 22 | 100 |  |
| Median size (IRQ) | 2 | (2-3) | 2 | (2-3) | NS | 3 | (2-4) | 3 | (2-4) | NS |
| Number of Cluster ≥3 | 221 | 44.5 | 93 | 42.1 | NS | 42 | 51.2 | 12 | 54.5 | NS |
| Median size (IRQ) | 4 | (3-5) | 3 | (3-4) | NS | 4 | (3-6) | 4 | (3-5) | NS |
| **Number of active clusters** | 120 | 24.1 | 31 | 14.0 | 0.002 | 26 | 31.7 | 3 | 13.6 | NS |
| Median size (IRQ) | 2 | (2-3) | 2 | (2-2) | NS | 2 | (2-3) | 2 | (2-2.5) | NS |
| Number of Cluster ≥3 | 36 | 7.2 | 1 | 0.5 | <0.0001 | 11 | 13.4 | 1 | 4.5 | NS |
| Median size (IRQ) | 3 | (3-4) | 3 | (3) | NS | 3 | (3-4) | 3 | (-) |  |
| **Number of clusters that suggest onward TDR** |  |  |  |  |  |  |  |  |  |  |
| Number of Cluster |  |  |  |  |  | 24 | 29.3 | 6 | 27.3 | NS |
| Median size (IRQ) | - | - | - | - |  | 3 | (3-4) | 3 | (3-3.75) | NS |
| Still active |  |  |  |  |  | 7 | 8.5 | - | - | NS |
| Median size (IRQ) | - | - | - | - |  | 2 | (2-2.5) |  |  |  |
|  |  |  |  |  |  |  |  |  |  |  |
| **Patients** |  |  |  |  |  |  |  |  |  |  |
| **Number in cohort clusters** |  |  |  |  |  |  |  |  |  |  |
| Number of cohort and controls | 1636 | 100 | 639 | 100 |  | 343 | 100 | 69 | 100 |  |
| Number of cohort patients | 1250 | 100 | 464 | 100 | NS | 146 | 100 | 33 | 100 | NS |
| Cluster ≥3 |  |  |  |  |  |  |  |  |  |  |
| Number of cohort and controls | 1084 | 66.3 | 383 | 59.9 | 0.005 | 263 | 76.7 | 49 | 71.0 | NS |
| Number of cohort patients | 828 | 66.2 | 273 | 58.8 | <0.0001 | 99 | 67.8 | 21 | 63.6 | NS |
| **Number in active clusters** |  |  |  |  |  |  |  |  |  |  |
| Number of cohort and controls | 302 | 18.5 | 63 | 9.9 | <0.0001 | 75 | 21.9 | 7 | 10.1 | 0.0306 |
| Number of cohort patients | 286 | 22.9 | 60 | 12.9 | <0.0001 | 40 | 27.4 | 4 | 12.1 | NS |
| Cluster ≥3 |  |  |  |  |  |  |  |  |  |  |
| Number of cohort and controls | 134 | 8.2 | 3 | 0.5 | <0.0001 | 43 | 12.5 | 3 | 4.3 | NS |
| Number of cohort patients | 128 | 10.2 | 3 | 0.6 | <0.0001 | 19 | 13.0 | 1 | 3.0 | NS |
| **Number in clusters that suggest onward TDR** |  |  |  |  |  |  |  |  |  |  |
| Cluster ≥3 |  |  |  |  |  |  |  |  |  |  |
| Number of cohort and controls | - | - | - | - |  | 95 | 27.7 | 21 | 63.6 | NS |
| Number of cohort patients | - | - | - | - |  | 72 | 49.3 | 14 | 42.4 | NS |
| Still active | - | - | - | - |  |  |  | - | - |  |
| Number of cohort and controls | - | - | - | - |  | 26 | 7.6 | - | - | NS |
| Number of cohort patients | - | - | - | - |  | 26 | 17.8 | - | - | NS |
